# Long‐term demography and spatial genetic structure reveal mechanisms of *Sassafras albidum* population persistence through clonality

**DOI:** 10.1002/ajb2.70215

**Published:** 2026-06-07

**Authors:** J. T. Michel, Jonathan P. Evans, Skyler J. Fox, Ashley B. Morris

**Affiliations:** ^1^ Department of Biology The University of the South Sewanee TN USA; ^2^ North Carolina Botanical Garden, University of North Carolina at Chapel Hill Chapel Hill NC USA; ^3^ Department of Biology Furman University Greenville SC USA

**Keywords:** basal sprouting, bud banks, clonal growth, demographic bottleneck, Lauraceae, regenerative ramet locations, Sassafras albidum, spatial genetic structure, tree clonality, vegetative regeneration

## Abstract

**Premise:**

Vegetative regeneration is a key mechanism of woody plant persistence in forest ecosystems, and the coupled roles of basal sprouting and clonal growth in shaping long‐term population dynamics have been understudied. Basal sprouting replaces stems at fixed ramet locations, whereas clonal growth via root suckering produces spatially distinct ramets that promote genet‐level persistence across space. *Sassafras albidum*, a clonal understory tree native to eastern North America, employs both mechanisms, making it an excellent system for evaluating how sprouting and clonality interact to structure populations.

**Methods:**

We combined over two decades of demographic data with spatially explicit microsatellite analyses to examine population structure, spatial genetic organization, and persistence mechanisms in an upland oak–hickory forest on the Cumberland Plateau, Tennessee.

**Results:**

Within a 1‐ha plot, we identified 31 genets, ranging from single‐ramet individuals to large clones exceeding 100 ramets and spanning more than 8000 m^2^. Clonal richness was low, indicating that most ramets were derived from clonal growth, yet clonal dominance was moderate to low, reflecting extensive intermingling of genets. Short and tall stems were spatially clustered and persistent through time, whereas intermediate stems were spatially and temporally unstable, revealing a demographic bottleneck. Genets differed markedly in their representation across size classes, indicating asynchronous demographic trajectories.

**Conclusions:**

We propose that the combined effects of basal sprouting and clonal growth generate persistent regenerative ramet locations, allowing genets to maintain advance regeneration across heterogeneous environments. These processes decouple stem turnover from genet turnover and facilitate long‐term persistence in resource‐poor upland forests.

Forest dynamics are strongly shaped by the persistence and longevity of woody plants, particularly in systems where recruitment from seed is episodic or unreliable (Connell and Slatyer, 1977; McCook, 1994). One major mechanism underlying persistence in woody species is vegetative regeneration, which allows individuals to survive disturbance, resource limitation, and prolonged suppression (Jenik, 1994; Bond and Midgley, 2001; Del Tredici, 2001). Among woody plants, vegetative regeneration is mediated through belowground bud banks—stores of dormant meristems capable of producing new shoots following stem mortality or release (Klimes, 2007; Ott et al., 2019).

Two common but functionally distinct forms of vegetative regeneration in forest trees are basal sprouting and clonal growth via root suckering. Basal sprouting refers to the production of new stems from dormant buds located at or near the base of an existing stem or root crown, resulting in stem replacement at a fixed ramet location (Bellingham and Sparrow, 2000; Del Tredici, 2001). This mechanism allows individual ramets to persist in place through repeated top‐kill, suppression, or partial mortality, often cycling between size classes as conditions change (Closset‐Kopp et al., 2007; Evans and Morris, 2016; Oldfield et al., 2021). In contrast, clonal growth via root suckering occurs when a genet produces spatially distinct ramets from lateral roots that are capable of physiological and spatial independence from the original stem (Silvertown and Lovett‐Doust, 1993; Del Tredici, 2001). Clonal growth allows genets to persist not only through time, but also across space, spreading risk of mortality among microsites and increasing the probability of encountering favorable resource conditions (Tanentzap et al., 2012).

Although basal sprouting and root suckering arise from the same underlying bud bank system, they differ fundamentally in their demographic consequences. Basal sprouting promotes persistence at a given ramet location, while clonal growth promotes genet‐level persistence through spatial expansion. Together, clonal growth and basal sprouting produce regenerative ramet locations (RRL), defined here as persistent ramet locations maintained through basal sprouting fueled by clustered, vascularized buds on long‐lived lateral roots, enabling long‐term genet persistence across heterogeneous environments (Figure 1). The regenerative ramet locations aid in decoupling stem turnover from genet turnover, allowing long‐lived genets to persist even when individual stems are short‐lived or recruitment from seed is infrequent.

**Figure 1:**
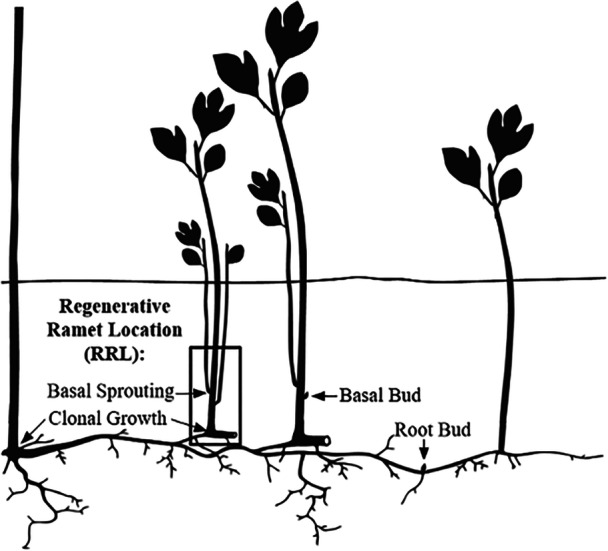
Graphical representation of *Sassafras albidum* vegetative regeneration. Regenerative ramet location is identified by the box. Root system is modeled after Duncan (1935).

Clonal growth produces a range of spatial architectures, from tightly clustered “phalanx” forms to widely dispersed “guerrilla” forms (Lovett‐Doust, 1981). These architectures influence not only local competition and resource acquisition, but also the spatial genetic structure (SGS) of populations (Dering et al., 2015). Genet clustering typically produces strong SGS, whereas intermingling of genets reduces SGS and may lower the probability of inbreeding (Chung et al., 2000; Dering et al., 2015). Clonality is widespread among woody angiosperms in eastern North America, including genera such as *Fagus* L., *Liquidambar* L., *Crataegus* L., *Sassafras* J. Presl, and *Robinia* L. (Loehle, 2000; Morris et al., 2014). However, no empirical studies have linked clonal architecture, SGS, and long‐term demography in this region.

*Sassafras albidum* (Nutt.) Nees (Lauraceae) provides a strong system for examining the interaction of basal sprouting and clonal growth because clonality via root suckering is a dominant and well‐documented feature of its population biology (Sullivan, 1993; Sharma et al., 2025). Across upland forests, root suckering accounts for most ramet production and often far exceeds recruitment from seed, making *S. albidum* also an excellent species for investigating genet persistence and spatial population structure (Ross et al., 1982; Blount, 1989). Despite this well‐documented capacity for clonal growth, the extent to which clonality structures population persistence, spatial genetic organization, and long‐term demography of *S. albidum* in mature upland forests remains poorly quantified. In particular, few studies have integrated long‐term demographic data with fine‐scale genetic data in woody plants to distinguish the relative contributions of basal sprouting and clonal expansion to population stability and persistence (Evans and Morris, 2016).

Here, we addressed this gap by combining over two decades of demographic monitoring with spatially explicit population genetic analyses of *S. albidum* in an upland oak–hickory forest on the Cumberland Plateau, Tennessee, United States. Our overarching research question was: What role does clonality play in the long‐term persistence of *S. albidum* populations? Specifically, we aimed to (1) identify and quantify genet identity and size within the population, (2) characterize clonal distribution at both ramet and genet spatial scales, (3) assess genet‐specific demographic patterns across size classes, and (4) evaluate how clonality and sprouting together contribute to long‐term population persistence under heterogeneous environmental conditions.

## MATERIALS AND METHODS

### Study species

*Sassafras albidum* is a dioecious, woody understory tree widely distributed across eastern North America and extending into the upper Midwest of the United States (Griggs et al., 1990). The species is insect‐pollinated (Gram and Sork, 2001), and its seeds are dispersed by birds, small mammals, and water (Sullivan, 1993). Seeds form a short‐term persistent seed bank and remain viable for more than 1 year (Hille Ris Lambers et al., 2005). Across its range, *S. albidum* occupies a variety of ecological settings; it can persist as either a dominant shrub or small tree in the understory of both mixed hardwood and pine forests (Ross et al., 1982). Population density and growth commonly increase following disturbance, including fire, canopy gaps (both natural and partial harvest gaps), and other forest management activities (Griggs et al., 1990; Grushecky and Fajvan, 1999; Brose, 2017; Alexander et al., 2021; Sharma et al., 2025). Although not generally considered a preferred browse species, likely due to the presence of safrole in its tissues, *S. albidum* is consumed by white‐tailed deer (Sample et al., 2022).

Across forested upland sites, root suckering in *S. albidum* accounts for the majority of ramet production and often far exceeds recruitment from seed (Ross et al., 1982; Blount, 1989; Sullivan, 1993; Sharma et al., 2025). The species forms extensive lateral root systems capable of rapid clonal expansion, with lateral growth rates reported to reach up to 1 m yr^−1^ (Bosela and Ewers, 1997). Excavations have shown that lateral roots naturally develop numerous additional buds as they grow through the soil, most of which become vascularized and connected to the primary xylem, indicating a high potential for repeated aboveground stem production from a single root segment, generally referred to in this species as root suckering (Bosela and Ewers, 1997). Individual ramets can remain physiologically connected at distances of at least 6 m for multiple years (Duncan, 1935; Crout, 2021), and these bud‐rich lateral root systems persist through repeated disturbance and canopy closure (Duncan, 1935; Ross et al., 1982; Blount, 1989). Clonal growth and persistence of small understory stems are especially common in xeric upland sites, where root suckering has been shown to account for up to 87% of ramet production (Blount, 1989).

### Study site

We studied *Sassafras albidum* in a 1 ha (100 × 100 m) plot of forest on the southwestern edge of the Cumberland Plateau, Tennessee, United States (35.219−85.962; 585 m a.s.l.) on the campus of the University of the South. The plot is in an upland forest typical of mixed‐oak communities on the southern Cumberland Plateau that is part of a long‐term study of plateau forest dynamics (Reid et al., 2008; Evans et al., 2019). Chestnut oak (*Quercus montana* Willd.) is the dominant tree in the plot, represented by 60% of all canopy individuals, followed in dominance by white oak (*Q. alba* L.) (15%), scarlet oak (*Q. coccinea* Wangenh.) (9%), and hickory (*Carya* spp. Nutt.) (6%). The subcanopy is composed of sourwood [*Oxydendrum arboreum* (L.) DC.], sassafras (*Sassafras albidum*), blackgum (*Nyssa sylvatica* Marshall), and red maple (*Acer rubrum* L.) (Evans et al., 2019). The soils in the study area are well drained and characteristic of the southern Cumberland Plateau, composed of Hartsells fine sandy loam and Muskingum stony fine sandy loam (Soil Survey Staff, 2019). This area is known for acidic and nutrient‐poor, thin soils (pH 4.5 ± 0.3) (Evans et al., 2019). The highest average temperatures are in July and August (28.0°C), with the lowest average temperature in January (−1.4°C). The average annual precipitation is 155 cm, with most precipitation between December and March (Evans et al., 2016). The forest is considered old second‐growth (Evans et al., 2019). According to university forest management and stand inventory records, the study site experienced very limited selective logging for *Quercus alba* in 1952, and for *Quercus coccinea*, *Nyssa sylvatica*, and *Carya* spp. in 1954, but has received no timber harvesting since that time.

### Population sampling

#### Size class A

One hectare was divided into a grid of 25 subplots, each 20 × 20 m, in 1999 (for further descriptions on plot and census design, see figure 2 of Oldfield et al., 2021). Four stratified random and four fixed circle plots (0.5 m radius) were marked for each subplot. *Sassafras albidum* “seedling” (<0.5 m tall) density within these circle plots was recorded and defined as size class A. The stratified random circle plots were surveyed in 1999, 2003–2010, 2013–2015, 2019, and 2025; the fixed circle plots were surveyed in 2006–2010, 2014–2015, 2019, and 2025.

#### Size classes B–F

All *S. albidum* stems >0.5 m tall were censused throughout the hectare (and with exact GPS coordinates starting in 2019). The nearest fixed circle plot was recorded for each individual, which allowed us to break the 20 × 20 m subplots into 10 × 10 m quads. Size classes were broken into 0.5–1 m (class B), >1–1.5 m (class C), >1.5–2 m (class D), >2–4 m (class E), >4 m (class F). Size classes B–F were censused in 2006–2008, 2010, 2019, and 2025. For the purposes of our study, we grouped size classes A and B as short stems, C–E as intermediate stems, and F as tall stems.

### Leaf collection protocol

In 2019, we took one leaf from each ramet >0.5 m tall within the hectare to extract DNA. If size class A ramets of *S. albidum* were present in a circle plot, we took one leaf from the closest ramet to the center of the circle plot. Leaves were also collected from ramets in plots outside the hectare: At four corner 20 × 20 m subplots of the hectare plot, we placed a flag 50 m outside the plot in the direction that the hectare border was oriented. We then collected a leaf from the nearest stem of *S. albidum* that was >0.5 m tall, but if no stem that large was found, a size A ramet closest to the flag was sampled. All sampled leaves were placed in silica in the field directly after collection.

### Development of microsatellite loci

Silica‐dried leaves were disrupted using a Qiagen TissueLyser II (Qiagen, Germantown, MD, USA). Genomic DNA from one individual of *Sassafras albidum* from Sewanee, Tennessee, was extracted using the Qiagen Dneasy Plant Pro Kit following the manufacturer's instructions. The sample was then submitted to the Evolutionary Genetics Core Facility, Cornell University, Ithaca, New York, United States to build a genomic library enriched for tetrameric repeats; the library was then sequenced on an Illumina MiSeq platform (San Diego, CA, USA). The sequenced library was mined for possible primer pairs using MSATCOMMANDER 1.0.3 (Faircloth, 2008). We screened 84 loci for their potential utility in this project using a fragment‐based approach. Sixteen individuals were included in the screen; eight were from four different subplots within the hectare plot (two from subplot 1, two from subplot 13, two from subplot 21, and two from subplot 25), seven from scattered locations across the Sewanee Domain (University of the South, Sewanee, Tennessee campus), and one from the campus of Furman University, Greenville, South Carolina.

For the PCR, we used the three‐primer approach of Schuelke (2000), in which a 17‐base tail (5′‐GTAAAACGACGGCCAGT‐3′) was added to the 5′ end of each forward primer and a 7‐base pigtail (5′‐GTTTCTT‐3′) was added to the 5′ end of each reverse primer. A third primer was synthesized to be identical to the 17‐base tail and was fluorescently labeled with a FAM fluorophore. The thermocycling conditions were described by Morris et al. (2016), and products were genotyped on an ABI SeqStudio (ThermoFisher Scientific, Waltham, MA, USA) at Furman University using GeneScan LIZ 500 size standard. All data were then scored using GeneMarker MTP software v. 2.6.0 (Softgenetics, State College, PA, USA).

### Microsatellite genotype‐by‐sequencing (GBS)

From the fragment‐based screening described above, 40 loci that were amplified consistently and cleanly were sent to the Evolutionary Genetics Core Facility of Cornell University for genotype‐by‐sequencing (GBS) as outlined by Fox and Morris (2025). Multiplex PCR included up to 20 loci per reaction, products were sequenced on an Illumina instrument, and custom python scripts written by the Cornell Bioinformatics Facility (https://bitbucket.org/cornell_bioinformatics/amplicon/src/master/) were used to call unique haplotypes (i.e., alleles) at each locus, treating individuals as tetraploid. Based on these initial results, we sent an additional 461 individuals of *S. albidum* for GBS using all 40 loci from the pilot. Upon receipt of the final data, we performed several additional quality control steps. We first confirmed that the top allele at each locus contained the expected tetranucleotide repeat. We then assessed depth of coverage for each allele at each locus and marked an individual genotype as missing if one or more alleles had less than five reads. Loci with ≥10% missing individuals were removed from the data set, resulting in the removal of 15 loci. Individuals with ≥10% missing loci were then removed from the data set, resulting in the removal of 43 individuals. The final, cleaned data set contained 22 loci and 418 individuals (402 within or adjacent to the hectare plot, 13 on the University of the South campus outside of the hectare plot, and 1 on the Furman University campus).

A genotype accumulation curve (GAC) was constructed using the R package poppr (Kamvar et al., 2014, 2015) and R version 4.5.2. (R Core Team, 2025) to determine whether the loci provided sufficient resolution for the current set of samples. The approach evaluates the number of unique multi‐locus genotypes (MLGs) for all possible combinations of 1 – *n* loci, where *n* is the total number of loci sampled. If a sufficient number of loci have been used to detect all MLGs in the sample set, the results of the GAC should reach an asymptotic curve.

### Clonal assignment

Ramets were assigned to gents using a distance‐based approach in the package genodive version 3.06 (Meirmans and Van Tienderen, 2004). As described in the program user manual, a genetic distance matrix is constructed, a threshold distance is chosen, and pairs of individuals with genetic distances below the chosen threshold are considered members of the same genet or clone. Because our genotypes are based on sequence data (not fragment length data), we chose the infinite allele mutation model (IAM), treating missing data as not counted. We evaluated a range of genetic distance thresholds (TH0–TH14) for determining clonal assignment (Table 1). Within the hectare study plot, for each distance threshold, we identified the number of genets, the largest genet by ramet count (largest *G*), and median and mean genet size by ramet count. Additionally, we calculated the proportion distinguishable (PD), which provides a summary of clonal richness and is calculated as the ratio of genets (*G*) to ramets (*N*; Ellstrand and Roose, 1987); values nearing 1 indicate sexual reproduction, while values nearing 0 indicate total clonality. For clonal richness we also calculated *R* as (*G* − 1)/(*N* − 1) (Dorken and Eckert, 2001), which is a modification of PD to adjust for small sample sizes. Based on field notes when leaves were sampled, we confirmed connectedness between two sampled ramets. We then calibrated our choice of genetic distance threshold based on which threshold first placed the connected ramets under the same genet, which happened to be TH6. All subsequent analyses were then based on a genetic distance threshold of TH6 unless otherwise noted. For visualization purposes, we associated colors with clonal assignments containing large numbers of ramets. Our selection of TH6 is further supported by the observation that 10 noted ramets with five‐lobed leaves belong exclusively to the pink clone (16), two ramets with four‐lobed leaves belong to the blue clone (30), and three fruiting ramets belong to the purple clone (4) at this threshold.

**Table 1:** *Sassafras albidum* clonal summary based on various genetic distance thresholds. Genets are denoted by *G*; clonal richness is denoted using PD, or proportion distinguishable, and is the ratio of genets to ramets. A modification of PD to account for small sample sizes is denoted by *R*.

Genetic distance threshold	*G*	No. single ramet *G*	No. multiramet *G*	Mean *G* by no. ramets	Median *G* by no. ramets	Largest *G* by no. ramets	PD	*R*
TH0	174	115	59	2.121	1	41	0.472	0.470
TH1	87	57	30	4.241	1	56	0.236	0.234
TH2	62	33	29	5.952	1	56	0.168	0.166
TH3	50	26	24	7.380	1	62	0.136	0.133
TH4	40	21	19	9.225	1	82	0.108	0.106
TH5	34	19	15	10.853	1	102	0.092	0.090
TH6	31	16	15	11.903	1	102	0.084	0.082
TH7	27	12	15	13.667	2	103	0.073	0.071
TH8	27	12	15	13.667	2	103	0.073	0.071
TH9	24	10	14	15.375	2	103	0.065	0.063
TH10	24	10	14	15.375	2	103	0.065	0.063
TH11	23	10	13	16.043	2	108	0.062	0.060
TH12	21	8	13	17.571	2	109	0.057	0.054
TH13	18	5	13	20.500	2.5	112	0.049	0.046
TH14	17	4	13	21.706	3	113	0.046	0.043

### Statistical analyses

Spatial extents of clones within the hectare were calculated using convex hull geometry in ArcGIS Pro and aligned with the methodology used by Ohsako (2010). R Studio (Posit Team, 2025) was used for all analyses unless otherwise stated. A Spearman's rank correlation test was performed to assess the connection between ramet count and spatial extents among clones. Mantel correlation was used for the genet‐identity autocorrelation (visualized in the correlogram) to account for our multivariate genetic data (Diniz‐Filho et al., 2013). Clonal dominance is a measure of clonal intermingling calculated as *D*_c_ = (*N*_S_ − 1)/(*N*_T_ − 1), where *N*_S_ is the number of ramets belonging to the genet of interest, and *N*_T_ is the number of total ramets (of all genets) sampled within the spatial extent of a clone (Ohsako, 2010). Using the method of Dering et al. (2015), we calculated mean *D*_c_ using clones with at least three ramets. For clonal dominance, a value approaching 1 indicates spatial exclusivity of a clone and a value approaching 0 indicates high levels of clonal intermingling (Ohsako, 2010). We used chi‐squared (*χ* ^2^) tests to assess the nonrandom distribution of mortality among genets and the nonrandom distribution of size class densities among genets.

Spatial clustering of *S. albidum* ramets was evaluated using Moran's *I*, calculated with an 8‐nearest‐neighbor spatial weights matrix. Moran's *I* was applied to ramet density for size A stem data collected in circle plots and to ramet density aggregated at the 10 × 10 m quad scale for size classes B–F. Spatial weights were derived from circle plot coordinates for size class A and from quad centroids for size classes B–F. Soil depth, soil moisture, and leaf litter depth were measured in circle plots and interpolated to continuous rasters using empirical Bayesian kriging in ArcGIS Pro. Mean values of these interpolated environmental variables and topographic variables (slope, aspect, and elevation) were then extracted to the quad scale using the zonal statistics tool in ArcGIS Pro for subsequent modeling. Relationships between ramet density and environmental variables were assessed using generalized linear mixed models (GLMMs) with count distributions appropriate to the dispersion characteristics of each dataset. Environmental variables were included as fixed effects, while quad and year were included as random intercepts to account for repeated spatial and temporal sampling. Negative binomial distributions were used in size classes B–F, and COM‐Poisson distributions were used for size class A. Spatiotemporal persistence in ramet density was evaluated by testing whether density at time *t* predicted density at time *t* + 1 within the same size class. This persistence was assessed by regressing density at time *t* + 1 on both local density and neighbor‐averaged density at time *t*, using the same 8‐nearest‐neighbor spatial weights matrix applied in the spatial autocorrelation analyses.

## RESULTS

### Clonality

#### Clonal composition

**Figure 2:**
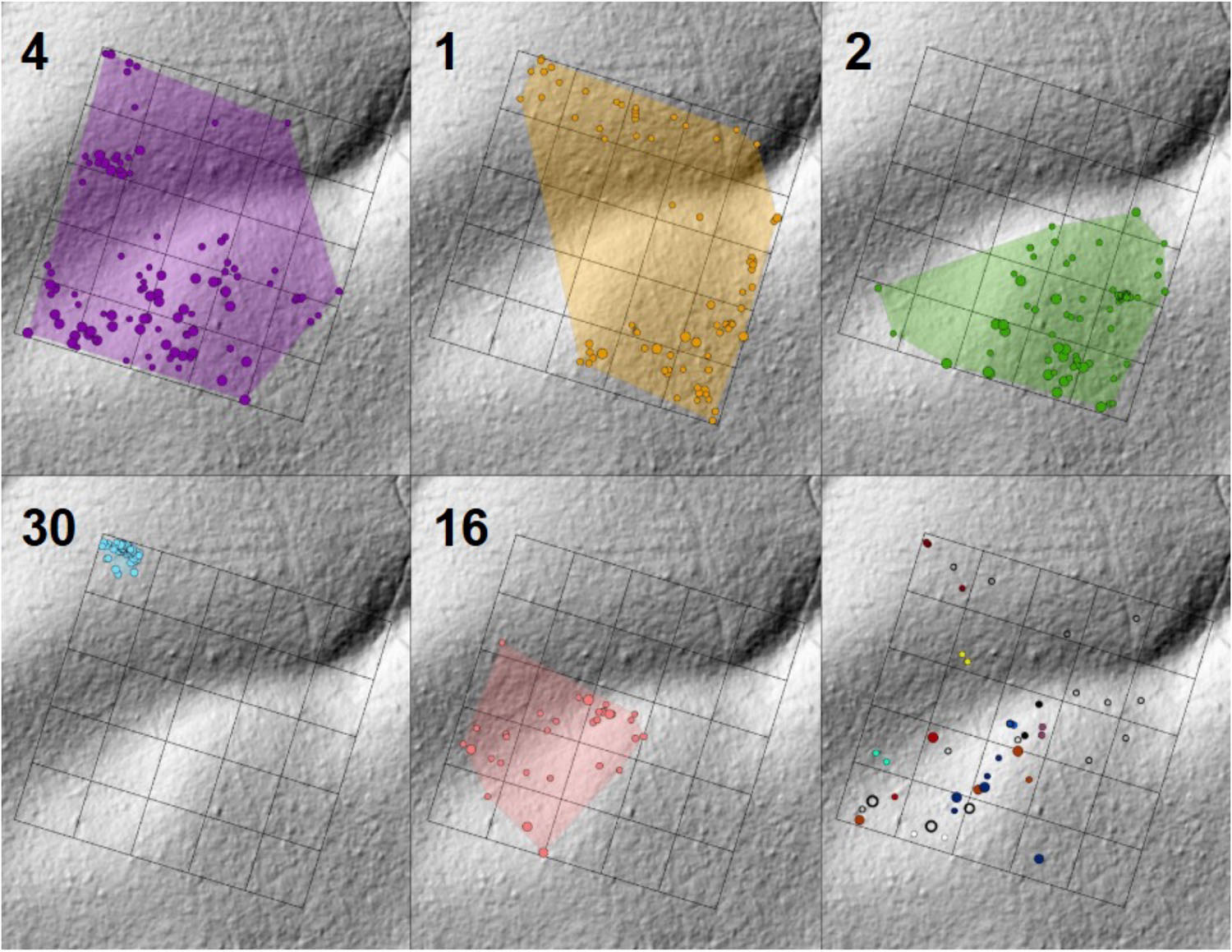
The five largest *Sassafras albidum* clones from a genetic distance threshold 6 in the hectare and their spatial extents used to calculate clonal dominance. The final frame (bottom right) shows smaller clones (identified by unique colors) and single‐ramet genets individuals (identified by open circles). Circle sizes are proportional to the ramet's size class.

**Figure 3:**
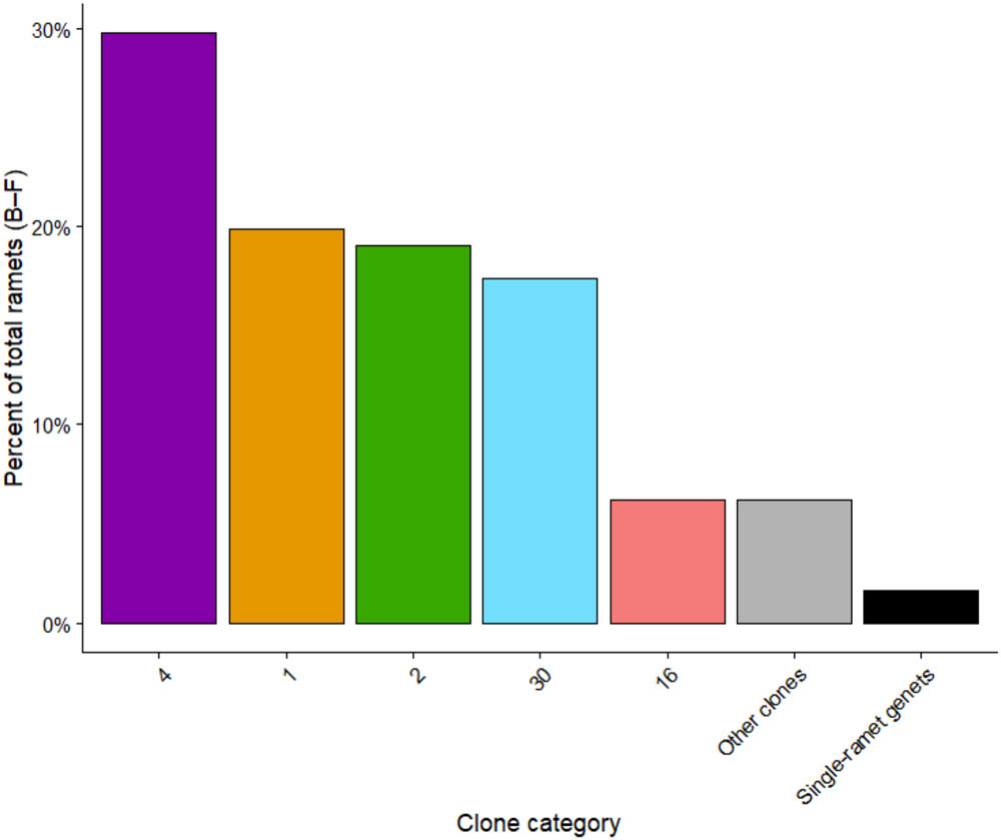
Percentage distribution of total *Sassafras albidum* ramets within size classes B–F by genet identity or category (denoted using color and clonal assignment number where applicable) within the hectare.

Given our sampling methods for size class A, it is important to note the potential underrepresentation of genetic diversity within this size class. Within the plot in 2019, we identified 31 genets (16 single‑ramet, 15 multiramet; Table 1). Multiramet genets ranged from 2 to 102 ramets and spanned 1 m^2^ to 8165 m^2^ (Table 2). Ramet count and spatial extent were strongly correlated (Spearman's *ρ* = 0.821, *P* < 0.001), with the blue clone (30) as an outlier due to its spatial extent being much smaller than expected based on its ramet counts (Figure 2, Table 2). Just over half of the genets were single ramets (Table 1), whereas a few dominant genets accounted for a disproportionate share of ramets in the hectare, with the fifth largest clone sharing the same percentage of ramets as all of the small clones combined (Figure 3). Clonal richness was low (PD = 0.084; *R* = 0.082), indicating that most ramets are derived from clonal growth.

**Table 2:** Total ramets, ramets by size class (A–F), and spatial extents of the *Sassafras albidum* clones within the hectare at TH6. Size classes were broken into <0.5 m tall (class A), >0.5–1 m tall (class B), >1–1.5 m tall (class C), >1.5–2 m tall (class D), >2–4 m tall (class E), >4 m tall (class F).

Clone	Total ramets	A	B	C	D	E	F	Spatial extent (m^2^)	Maximum distance (m)
4 (Purple)	102	32	26	4	2	2	36	8165	215
1 (Gold)	76	28	37	4	1	2	4	6975	167
2 (Green)	73	27	25	3	1	0	17	4158	123
30 (Blue)	43	1	23	12	5	2	0	97	60
16 (Pink)	33	18	10	0	0	4	1	2475	73
9	6	4	0	0	0	1	2	409	37
15	4	1	0	0	0	2	1	307	59
36	3	1	2	0	0	0	0	1	20
19	2	0	2	0	0	0	0	2	1
20	2	0	2	0	0	0	0	23	12
21	2	0	2	0	0	0	0	6	3
23	2	2	0	0	0	0	0	21	11
27	2	1	0	0	0	0	1	48	24
28	2	2	0	0	0	0	0	6	3
33	2	2	0	0	0	0	0	9	5

*Notes*: Maximum distance was calculated using ramets outside the hectare study plot if they were present. The five largest clones are listed with the color that was used for mapping (Figure 2).

#### Spatial genetic structure (SGS)

**Figure 4:**
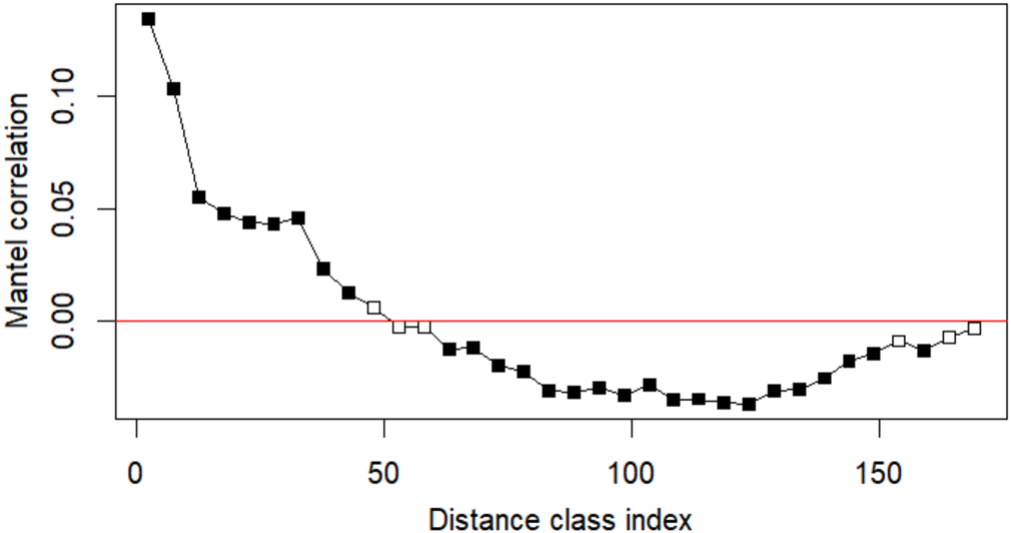
Correlogram of genetic similarity by distance class. We constructed 34 distance classes of 5 m to account for all possible ramet pairs. Filled squares indicate significant genet correlation for that distance class, while open squares indicate nonsignificant correlations.

Genet‑identity autocorrelation (34 distance classes of 5 m) indicated significant positive spatial genetic structure (SGS) ≤ 45 m, significant negative SGS from 65 to 150 m, and largely nonsignificant values at 155–170 m (Figure 4). This nonsignificance is likely due to small sample size within these later distance classes and could be skewed toward nonsignificance due to the large clones that extend throughout most of the plot (Figure 2). Overall, spatial genetic structure was strong at short to medium distances (up to 45 m). Among the five largest clones, clonal dominance was low except for the blue clone (30) with a small spatial extent (Table 3), though we observed that many clones, including blue, have spatial extents beyond the bounds of our plot (Appendix S1; Table 2). Clones showed a considerable level of intermingling, with the mean *D*_c_ = 0.4 at TH6. The genet‐identity autocorrelation and mean clonal dominance suggest that spatial genetic structure is strong for limited extents, while moderate to high levels of intermingling within these areas can simultaneously occur (Figure 2, Table 3).

**Table 3:** Clonal dominance for each *Sassafras albidum* clone with ≥3 ramets by genetic distance threshold 6 (GD TH 6).

Clone	Spatial extent (m^2^)	Total ramets within extent	Total target clone ramets within extent	Clonal dominance
**GD TH 6**				
4 (Purple)	8165.198	319	102	0.318
1 (Gold)	6975.009	271	76	0.278
2 (Green)	4158.312	212	73	0.341
30 (Blue)	97.048	51	43	0.840
16 (Pink)	2475.063	88	33	0.368
9	409	28	6	0.185
15	307	18	4	0.176
36	1	4	3	0.667

*Note*: Color (and number associated with TH6 clonal assignment) corresponds to clonal representation in Figure 3.

#### Demographic assessments by clonality

**Figure 5:**
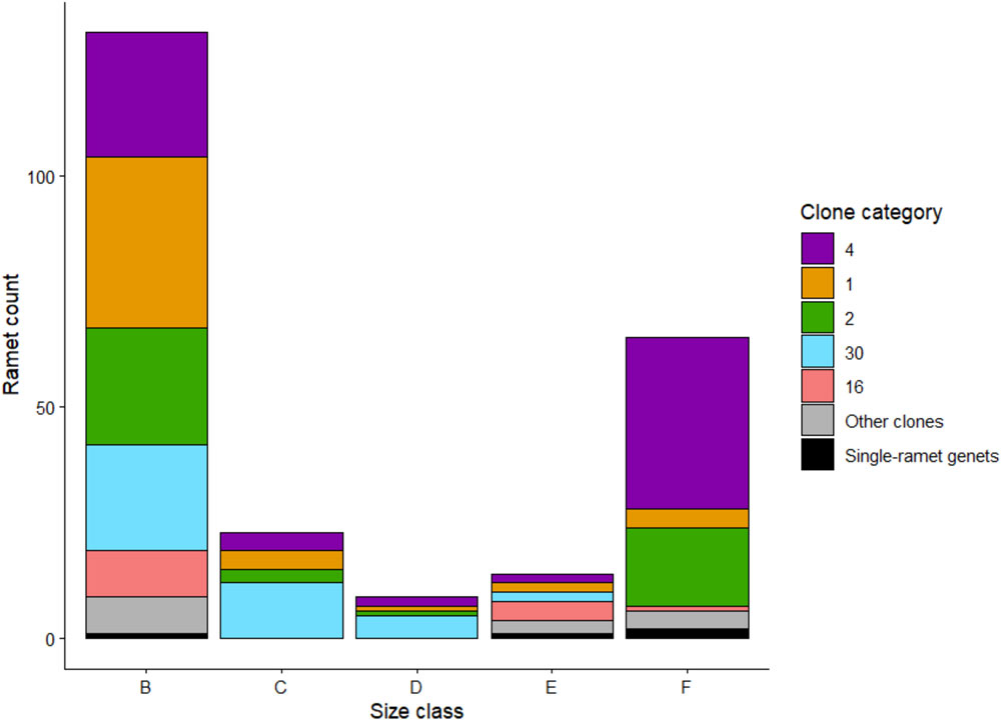
Ramet density distribution of vertical size classes A–F by genet category within the hectare, including the five largest clones. Size classes were broken into <0.5 m tall (class A), >0.5–1 m tall (class B), >1–1.5 m tall (class C), >1.5–2 m tall (class D), >2–4 m tall (class E), >4 m tall (class F).

From 2019 to 2025, mortality among size classes D–F was proportional to each genet's ramet abundance (*χ* ^2^ = 6.11, df = 11, *P* = 0.87). In contrast, genet by size‑class (B–F) frequencies were significantly nonrandom (*χ* ^2^ = 150.95, df = 60, *P* < 0.001; Figure 5), indicating unequal distribution of clones among size classes. Residuals identified specific patterns: purple (4) underrepresented in B and overrepresented in F; gold (1) the reverse; blue (30) overrepresented in C and D and absent from F; pink (16; and several smaller genets) overrepresented in E due to the relatively low abundance of ramets in this size class (Table 4).

**Table 4:** *Sassafras albidum* clonal ramet density over‐ and underrepresentation by size class. Size classes were broken into <0.5 m tall (class A), >0.5–1 m tall (class B), >1–1.5 m tall (class C), >1.5–2 m tall (class D), >2–4 m tall (class E), >4 m tall (class F).

Clone	Size class	Residual
4 (Purple)	B	–3.379
	F	5.603
1 (Gold)	B	3.564
	F	–3.234
30 (Blue)	C	4.635
	D	3.084
	F	–4.320
16 (Pink)	E	3.577
9	E	2.057
14	E	4.044
15	E	4.545

*Notes*: Chi‐squared residual values >2 mark overrepresentation of that clone in that size class and residuals <–2 mark underrepresentation of that clone in that size class. Residuals of nonsignificant overrepresentation and underrepresentation are not shown.

### Demography

#### Spatial clustering and environmental factors

**Figure 6:**
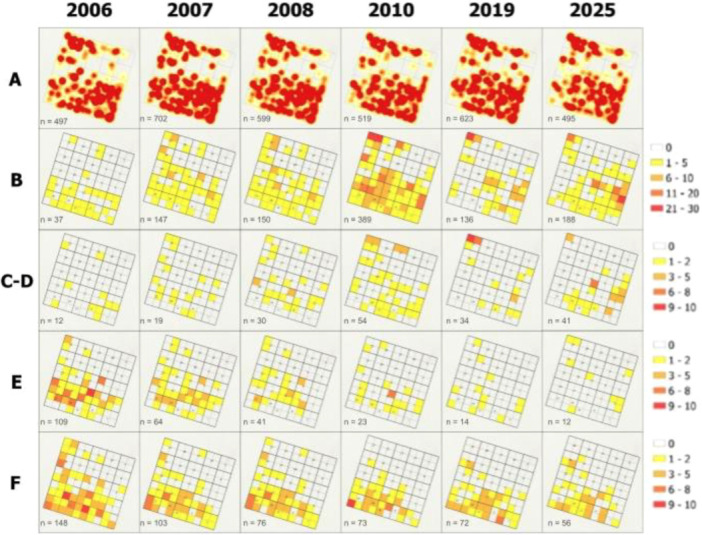
Heat map of *Sassafras albidum* density by size class and year. Size class A is represented by a heat map with densities populated from the eight circle plots in each 20 × 20 m. Size classes B–F are populated as complete densities per 10 × 10 m. Size class B is set on a different scale than size classes C–F for their variation in relative abundance. Size classes C and D have been combined here for their functional similarity. Total number of stems are identified for each size class and year at the bottom left corner of each cell.

For short and tall stems (A, B, and F), there is continuously significant spatial clustering of *S. albidum* density across census periods, whereas in the intermediate stems (C–E), there is not continuously significant spatial clustering (Figure 6; Appendix S2). Across size classes, environmental gradients varied in their correlation with *S. albidum* density (Appendix S3). Size class A stem densities were significantly influenced by less‐steep slopes, north‐facing areas, and higher elevation. Elevation and soil moisture had a significant negative relationship (Spearman's *ρ* = –0.176, *P* < 0.001). Soil depth had a marginal negative association with the density of A stems. Size B stems showed significant densities along flatter, northwest‐facing areas. High elevation was marginally associated with higher densities of B stems, while greater soil depth and lower soil moisture were also significantly correlated with stem densities in this size class. For both size C and D stems, densities were significantly correlated with west‐facing, greater soil depth, and lower soil moisture areas. For size classes E and F, high elevation was the only significant environmental variable (for significance values of environmental factors see Appendix S3).

#### Ramet persistence

**Figure 7:**
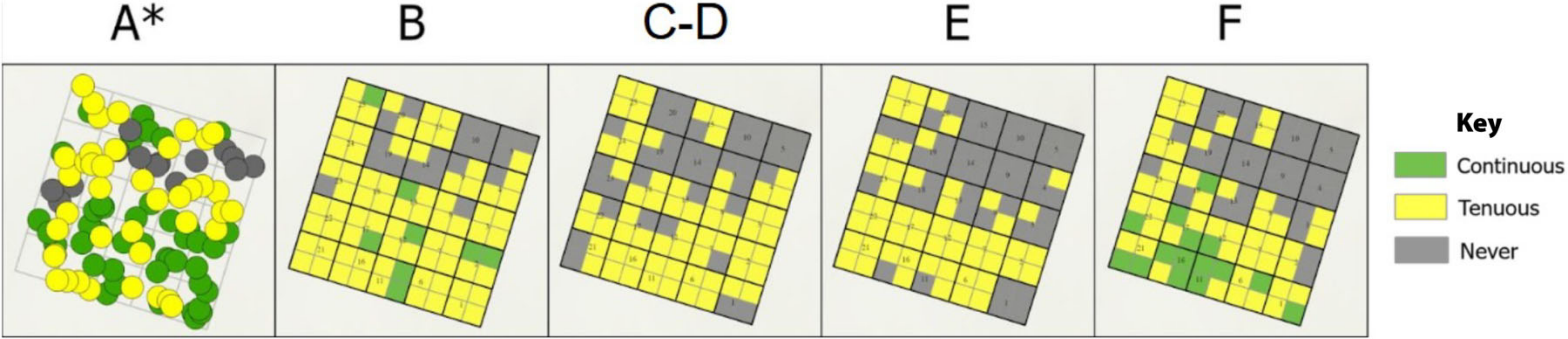
*Sassafras albidum* occupancy by size class. “Continuous” (green) marks which circle plots or quads always had a stem density in each census. “Tenuous” (yellow) marks that circle plots or quads had stems in at least one but not all census periods; “Never” (gray) indicates circle plots or quads that never had stems in any census. Size classes C and D have been combined here for their functional similarity. *Four stratified random circle plots were used for size class A in 1999, 2003–2010, 2013–2015, 2019, and 2025. Size classes B–F used data from 10 × 10 m quads in 2006–2008, 2010, 2019, and 2025.

Given the variable intervals between census periods, we were able to show both short‐ (2006–07, 2007–08, 2008–10) and long‐term (2010–19, 2019–25) persistence strategies among *S. albidum* densities. Density in short and tall stems were significantly correlated with the density at the next census in the same size class for both the short‐ and long‐term in the same 10 × 10 m quad locations, whereas significance for all intermediate stems in predicting *S. albidum* density was inconsistent (Appendix S4). For stems in size C, density values were correlated every year, except for the short‐term interval of 2006–07. For stems in size D, density values were only significantly correlated between the long‐term interval of 2019 and 2025. For stems in size E, density values were not correlated between 2010 and 2019, but were highly significant for every other interval (Appendix S4). Short and tall stems maintained their density levels throughout all census periods and had consistent occupancy in some quads, whereas intermediate stems appeared more ephemeral in their densities over time, with no quads having consistent occupancy throughout all census periods (Figure 7; Appendix [Link], [Link]).

## DISCUSSION

We demonstrated that clonality is a central mechanism structuring the long‐term persistence of *Sassafras albidum* populations in upland oak–hickory forests of the Cumberland Plateau. By integrating more than two decades of demographic data with spatially explicit genetic analyses, we showed that persistence emerges from the combined but functionally distinct roles of basal sprouting and clonal growth via root suckering. Together, these processes decouple stem turnover from genet turnover, allowing long‐lived genets to persist despite substantial temporal variability in stem abundance and size structure. Similar decoupling has been reported for other clonal woody species, including *Populus alba* L. (Dering et al., 2015), *Robinia pseudoacacia* L. (Chang et al., 1998), and *Asimina triloba* (L.) Dunal. (Hosaka et al., 2008), but few studies have examined these processes using long‐term demographic and genetic data within mature upland forests.

### Clonal growth and spatial genetic structure

Within our hectare plot, we identified 31 genets, with most ramets derived from a relatively small number of large clones. Low clonal richness is consistent with studies of clonal woody species in environments where sexual recruitment is limited or episodic (Eriksson and Fröborg, 1996), such as *Robinia pseudoacacia* (Chang et al., 1998) and *Betula humilis* Marshall (Bona et al., 2019). Despite the presence of very large clones—some exceeding 100 ramets and spanning more than 8000 m^2^—clonal dominance was moderate to low, indicating substantial intermingling of genets rather than exclusive spatial occupation.

This pattern contrasts with strongly clustered genet structures reported for some clonal trees, including *Quercus laevis* Walter, *Q. margaretta* (Ashe) Small (Berg and Hamrick, 1994), and *Q. chrysolepis* Liebm. (Montalvo et al., 1997). Our results indicate that *S. albidum* more closely resembles intermingled architectures observed in *Prunus avium* (L.) L. (Vaughan et al., 2007), *Rhus javanica* L. (Chung et al., 2000), *Tilia cordata* Mill. (Erichsen et al., 2019), and some populations of *Populus tremuloides* Michx. (Namroud et al., 2005). Intermingled clonal architectures are commonly associated with heterogeneous environments and patchy disturbance regimes (Namroud et al., 2005), conditions characteristic of upland forests on the Cumberland Plateau.

The observed spatial genetic structure (positive autocorrelation at short distances followed by negative autocorrelation at larger distances) suggests that population structure reflects long‐term lateral expansion of overlapping genets rather than recent recruitment. Similar spatial genetic patterns have been attributed to cumulative clonal growth in *Populus alba* (Dering et al., 2015) and *Sorbus torminalis* (L.) Crantz (Hoebee et al., 2006). Given documented rates of lateral root expansion in *S. albidum* (Bosela and Ewers, 1997), the largest clones in this study are likely centuries old, reinforcing the conclusion that genet persistence operates on much longer timescales than stem turnover.

### Basal sprouting and persistence at fixed ramet locations

While clonal growth structures population organization at the genet level, basal sprouting functions primarily as a mechanism of persistence at fixed ramet locations. Short stems were abundant, spatially clustered, and temporally stable across census periods, consistent with long‐term advance regeneration maintained through repeated stem replacement (Hutchinson et al., 2012). Comparable patterns have been reported in other sprouting understory trees and shrubs, including *Prunus serotina* Ehrh. (Closset‐Kopp et al., 2007) and *Quercus montana* (Oldfield et al., 2021) where ramets persist for decades by cycling through size classes under changing light conditions.

In *S. albidum*, basal sprouting allows ramets to survive prolonged suppression and canopy closure without spatial relocation. However, unlike species that rely primarily on sprouting at fixed locations, *S. albidum* also employs extensive clonal growth, allowing persistence to operate simultaneously at multiple spatial scales. This dual strategy increases the likelihood that at least some ramets within a genet will encounter favorable conditions for growth.

### Demographic bottlenecks and genet‐level heterogeneity

A key result of this study is the identification of a demographic bottleneck at intermediate size classes, where stem densities were spatially and temporally unstable. Intermediate stems neither persisted at fixed locations nor transitioned reliably into larger size classes. Additional bottlenecks have been documented in *Quercus* spp. under fire suppression (Cho and Boerner, 1991; Nowacki and Abrams, 2008) and in understory shrubs such as *Lindera triloba* Hook.f & Thomson ex Meisn. (Matsushita et al., 2010), where mortality risk is highest during the transition from physiological integration to independence.

Genets differed markedly in their representation across size classes, indicating asynchronous demographic trajectories among clones. Similar genet‐level heterogeneity has been observed in *Robinia pseudoacacia* (Chang et al., 1998) and *Magnolia stellata* (Siebold & Zucc.) Maxim. (Setsuko et al., 2004) and likely reflects differences in microsite conditions, disturbance history, and clone age. Our results show that such heterogeneity persists even within a relatively small spatial extent and is structured by clonal architecture.

### Regenerative ramet locations and potential mechanisms mediating persistence

The combined action of basal sprouting and clonal growth gives rise to regenerative ramet locations (RRL), which represent sites of persistent stem replacement distributed across the landscape. In *S. albidum*, long‐lived lateral root systems bearing clustered, vascularized buds allow repeated stem replacement at fixed locations while simultaneously generating new ramet locations through continued root extension (Duncan, 1935; Ross et al., 1982; Blount, 1989; Bosela and Ewers, 1997). Together, these processes create a spatially distributed network of advance regeneration that promotes long‐term genet persistence in heterogeneous environments. Notably, only one genet died—a single‐ramet genet, further supporting the understanding that clonality helps to buffer against the loss of genetic individuals in woody plants (Hoebee et al., 2006; Matsushita et al., 2010; Dering et al., 2015).

Clonal integration could further enhance the persistence value of RRL by maintaining physiological connectivity among ramets. Through shared root systems, resources acquired in favorable microsites may subsidize suppressed or newly initiated ramets elsewhere within a genet, reducing mortality risk during periods of low light or environmental stress. Similar benefits of clonal integration have been documented in other clonal woody species, including *Populus tremuloides*, *Robinia pseudoacacia*, and *Asimina triloba*, where integration buffers demographic variability and supports long‐term persistence (Zhang et al., 2006; Pinno and Wilson, 2014; Hosaka et al., 2016).

Although we did not directly quantify physiological integration among ramets, previous work indicates that *S. albidum* can develop extensive networks of belowground root connectivity that can persist for at least 7 years (Duncan, 1935; Blount, 1989). The spatial distribution of ramets across environmental gradients in our study (particularly elevation and associated soil moisture differences) suggests that favorable placement of lateral root growth (“foraging”; Hutchings and de Kroon, 1994; Evans and Cain, 1995) may contribute to genet persistence in resource‐poor upland environments (Evans et al., 2019). Clonal growth is especially prevalent in xeric sites (Ross et al., 1982), and the predominance of clonally produced ramets in our population, including concentrations at higher elevations with reduced soil moisture, aligns with earlier observations (Blount, 1989).

In *S. albidum*, photosynthetically derived carbon can be incorporated into both higher‐order and first‐order roots more than a year after root initiation (Adams and Eissenstat, 2014), indicating sustained investment in long‐lived root systems. *Sassafras albidum* also forms associations with arbuscular mycorrhizal fungi (Sharma et al., 2025), which enhance inorganic nutrient uptake (Phillips et al., 2013). Nutrient foraging through mycorrhizal mutualisms has been proposed as an advantageous strategy for thick‐rooted species (Eissenstat et al., 2015; Chen et al., 2018), such as *S. albidum* (Adams et al., 2013). Although the physiological mechanisms underlying prolonged carbon incorporation into *S. albidum* roots remain unresolved (Adams and Eissenstat, 2014), continued carbon investment may support mycorrhizal associations that enhance nutrient acquisition across expansive clonal root networks (Gavito and Olsson, 2003).

Together, these observations suggest that long‐lived, integrated root systems may allow *S. albidum* genets to maintain persistent regenerative ramet locations along favorable environmental gradients, while sustained carbon allocation and mycorrhizal symbioses could enhance nutrient foraging across heterogeneous and resource‐limited upland soils. Through these interacting belowground processes, clonality, integration, and mycorrhizal mutualisms jointly contribute to the decoupling of stem‐level dynamics from long‐term genet persistence.

### Management implications

Our results indicate that long‐term persistence of *S. albidum* populations depends on the maintenance of belowground legacies, including lateral root systems, bud banks, and regenerative ramet locations (RRL), rather than on the survival of tall stems alone. Persistence in this study was linked to repeated basal sprouting and clonal replacement at fixed ramet locations, indicating that disturbances causing aboveground top‐kill do not necessarily result in immediate genet loss. Disturbances that leave root systems intact—such as low‐intensity fire or limited canopy disturbance—are therefore more likely to sustain genet persistence (Grushecky and Fajvan, 1999; Sharma et al., 2025), whereas intensive mechanical harvesting or soil disturbance may disrupt lateral roots and bud banks, leading to genet loss despite high post‐disturbance stem densities.

Our findings are particularly relevant in the context of laurel wilt disease caused by the fungus *Raffaelea lauricola* carried by the redbay ambrosia beetle (*Xyleborus glabratus* Eichhoff; Cameron et al., 2015; Randolph, 2017; Crout, 2021; Ward and Riggins, 2023), which causes rapid mortality of large stems in members of the Lauraceae but does not immediately eliminate belowground structures (Evans et al., 2014). In *Persea borbonia* (L.) Spreng. (redbay), laurel wilt results in near‐complete canopy mortality, while basal sprouting commonly persists after stem death; however, repeated browsing by white‐tailed deer often prevents sprouts from escaping the browse zone, leading to long‐term genet loss despite prolific sprouting (Fraedrich et al., 2008; Evans et al., 2014). The spread of laurel wilt from redbay populations to *S. albidum* has resulted in *S. albidum* now being impacted across a large portion of its range (Georgia Forestry Commission, 2020), including populations adjacent to our study site. Our results suggest that *S. albidum* may follow a similar trajectory under laurel wilt pressure: Although basal sprouting and clonal growth may initially enable persistence after tall stem mortality, sustained browse pressure could prevent progression through the intermediate‐size bottleneck and severely constrain long‐term genet persistence.

More broadly, these findings highlight the importance of distinguishing between stem mortality and genet mortality when evaluating pathogen impacts and management responses because apparent recovery based on sprout abundance alone may mask long‐term losses of genetic individuals.

## CONCLUSIONS

We demonstrated that long‐term persistence of *Sassafras albidum* populations in upland forests is driven by the combined action of basal sprouting and clonal growth via root suckering. By combining long‐term demographic data with spatially explicit genetic analyses, we showed that population structure is characterized by low clonal richness, extensive intermingling of genets, and marked heterogeneity in genet representation across size classes. Short and tall stems were spatially persistent, whereas intermediate‐size stems exhibited spatial and temporal instability, revealing a demographic bottleneck that constrains progression through the size structure.

Our results indicate that basal sprouting promotes persistence at fixed ramet locations, while clonal growth expands genets across space, together producing regenerative ramet locations that buffer demographic variability over time. These processes decouple stem turnover from genet turnover and allow populations to persist in resource‐poor upland environments despite limited recruitment from seed. By explicitly distinguishing between basal sprouting and clonal growth, this study highlights the importance of genet‐level processes in structuring understory tree populations and underscores clonality as a key mechanism of long‐term population persistence in temperate forests.

## AUTHOR CONTRIBUTIONS

J.P.E. and A.B.M. conceived and designed the study. All authors contributed to fieldwork, material preparation, and data collection. S.J.F. conducted molecular work for the study. J.T.M. conducted the statistical analyses and led manuscript writing, with all authors contributing to revisions. All authors read and approved the final manuscript.

## Supporting information


**Appendix S1:** 3D video animation of the spatial distribution of *Sassafras albidum* ramets in size classes B–F and their genet identity within and outside of the study plot. Genet identity is visualized by color, using the genetic distance threshold TH6. Ramet size class is visualized by column height.

**Appendix S2:** *Sassafras albidum* ramet density clustering by size class and census period using Moran's *I* spatial autocorrelation with 8‐nearest‐neighbor spatial weights matrix.

**Appendix S3:** *Sassafras albidum* ramet density along environmental gradients using generalized linear mixed models.

**Appendix S4:** Spatiotemporal persistence of *Sassafras albidum* ramet density by size class and census interval using spatial regression with an 8‐nearest‐neighbor spatial weights matrix.

## Data Availability

All data sets used in this study are deposited in Figshare: multiple thresholds for clonal assignment, https://doi.org/10.6084/m9.figshare.31366354; spatially explicit ramet density, https://doi.org/10.6084/m9.figshare.31366429; spatially explicit environmental data, https://doi.org/10.6084/m9.figshare.31366438; spatially explicit ramet density (size A), https://doi.org/10.6084/m9.figshare.31366456.
