# Supplementary material for: Long‐term demography and spatial genetic structure reveal mechanisms of Sassafras albidum population persistence through clonality
Source: Am J Bot. 2026 Jun 7;113(6):e70215. doi: 10.1002/ajb2.70215 (PMC13280968; doi:10.1002/ajb2.70215)
Supplement: Supplementary file 2 — Appendix S2: Sassafras albidum ramet density clustering by size class and census period using Moran's I spatial autocorrelation with 8‐nearest‐neighbor spatial weights matrix. [file AJB2-113-e70215-s001.docx]

**Appendix S2.** *Sassafras albidum* ramet density clustering by size class and census period using Moran’s *I* spatial autocorrelation with 8-nearest-neighbor spatial weights matrix.

| Size class | Year | *I* | *P* |
| --- | --- | --- | --- |
| A | 1999 | 0.109 | 0.005** |
|  | 2003 | 0.270 | <0.001*** |
|  | 2004 | 0.226 | <0.001*** |
|  | 2005 | 0.277 | <0.001*** |
|  | 2006 | 0.229 | <0.001*** |
|  | 2007 | 0.184 | <0.001*** |
|  | 2008 | 0.243 | <0.001*** |
|  | 2009 | 0.298 | <0.001*** |
|  | 2010 | 0.175 | <0.001*** |
|  | 2013 | 0.265 | <0.001*** |
|  | 2014 | 0.254 | <0.001*** |
|  | 2015 | 0.259 | <0.001*** |
|  | 2019 | 0.160 | <0.001*** |
|  | 2025 | 0.179 | <0.001*** |
| B | 2006 | 0.285 | <0.001*** |
|  | 2007 | 0.072 | 0.040* |
|  | 2008 | 0.068 | 0.050* |
|  | 2010 | 0.166 | <0.001*** |
|  | 2019 | 0.109 | 0.002** |
|  | 2025 | 0.227 | <0.001*** |
| C | 2006 | 0.074 | 0.029* |
|  | 2007 | -0.066 | 0.898 |
|  | 2008 | -0.058 | 0.853 |
|  | 2010 | 0.099 | 0.008** |
|  | 2019 | 0.100 | 0.003** |
|  | 2025 | 0.120 | 0.002** |
| D | 2006 | 0.051 | 0.077 |
|  | 2007 | -0.004 | 0.441 |
|  | 2008 | 0.013 | 0.298 |
|  | 2010 | 0.051 | 0.081 |
|  | 2019 | 0.032 | 0.041* |
|  | 2025 | -0.057 | 0.869 |
| E | 2006 | 0.196 | <0.001*** |
|  | 2007 | 0.152 | <0.001*** |
|  | 2008 | 0.166 | <0.001*** |
|  | 2010 | 0.029 | 0.139 |
|  | 2019 | -0.004 | 0.449 |
|  | 2025 | -0.002 | 0.431 |
| F | 2006 | 0.250 | <0.001*** |
|  | 2007 | 0.390 | <0.001*** |
|  | 2008 | 0.316 | <0.001*** |
|  | 2010 | 0.341 | <0.001*** |
|  | 2019 | 0.339 | <0.001*** |
|  | 2025 | 0.287 | <0.001*** |
