# Supplementary material for: Long‐term demography and spatial genetic structure reveal mechanisms of Sassafras albidum population persistence through clonality
Source: Am J Bot. 2026 Jun 7;113(6):e70215. doi: 10.1002/ajb2.70215 (PMC13280968; doi:10.1002/ajb2.70215)
Supplement: Supplementary file 3 — Appendix S3: Sassafras albidum ramet density along environmental gradients using generalized linear mixed models. [file AJB2-113-e70215-s003.docx]

**Appendix S3.** *Sassafras albidum* ramet density along environmental gradients using generalized linear mixed models.

| Size class | Variable | Estimate | Standard error | *z* | *P* |
| --- | --- | --- | --- | --- | --- |
| A | Slope | -0.949 | 0.245 | -3.870 | <0.001*** |
|  | Elevation | 0.450 | 0.208 | 2.164 | 0.030* |
|  | Northness | 0.506 | 0.167 | 3.026 | 0.002** |
|  | Eastness | -0.272 | 0.192 | -1.415 | 0.157 |
|  | Soil depth | -0.315 | 0.170 | -1.851 | 0.064 |
|  | Soil moisture | 0.054 | 0.149 | 0.364 | 0.716 |
|  | Leaf litter depth | -0.231 | 0.153 | -1.515 | 0.130 |
| B | Slope | -0.466 | 0.169 | -2.757 | 0.006** |
|  | Elevation | 0.285 | 0.154 | 1.856 | 0.064 |
|  | Northness | 0.281 | 0.123 | 2.286 | 0.022* |
|  | Eastness | -0.564 | 0.147 | -3.828 | <0.001*** |
|  | Soil depth | 0.534 | 0.145 | 3.698 | <0.001*** |
|  | Soil moisture | -0.303 | 0.119 | -2.541 | 0.011* |
|  | Leaf litter depth | -0.230 | 0.137 | -1.680 | 0.093 |
| C | Slope | -0.438 | 0.252 | -1.740 | 0.082 |
|  | Elevation | 0.355 | 0.225 | 1.575 | 0.115 |
|  | Northness | 0.050 | 0.173 | 0.288 | 0.773 |
|  | Eastness | -0.515 | 0.220 | -2.334 | 0.020* |
|  | Soil depth | 0.619 | 0.215 | 2.885 | 0.004** |
|  | Soil moisture | -0.451 | 0.179 | -2.513 | 0.012* |
|  | Leaf litter depth | -0.271 | 0.196 | -1.382 | 0.167 |
| D | Slope | -0.385 | 0.325 | -1.183 | 0.237 |
|  | Elevation | 0.390 | 0.269 | 1.449 | 0.147 |
|  | Northness | 0.123 | 0.212 | 0.580 | 0.562 |
|  | Eastness | -0.640 | 0.265 | -2.416 | 0.016* |
|  | Soil depth | 0.567 | 0.252 | 2.245 | 0.025* |
|  | Soil moisture | -0.618 | 0.238 | -2.595 | 0.009** |
|  | Leaf litter depth | 0.194 | 0.233 | 0.833 | 0.405 |
| E | Slope | 0.037 | 0.312 | 0.119 | 0.905 |
|  | Elevation | 1.090 | 0.297 | 3.673 | <0.001*** |
|  | Northness | 0.223 | 0.207 | 1.075 | 0.282 |
|  | Eastness | -0.477 | 0.253 | -1.881 | 0.060 |
|  | Soil depth | 0.492 | 0.255 | 1.930 | 0.054 |
|  | Soil moisture | -0.212 | 0.225 | -0.941 | 0.347 |
|  | Leaf litter depth | 0.077 | 0.287 | 0.266 | 0.790 |
| F | Slope | 0.023 | 0.238 | 0.099 | 0.922 |
|  | Elevation | 1.679 | 0.233 | 7.219 | <0.001*** |
|  | Northness | 0.202 | 0.162 | 1.252 | 0.211 |
|  | Eastness | -0.197 | 0.199 | -0.989 | 0.323 |
|  | Soil depth | -0.056 | 0.194 | -0.287 | 0.774 |
|  | Soil moisture | 0.186 | 0.171 | 1.091 | 0.275 |
|  | Leaf litter depth | 0.156 | 0.189 | 0.827 | 0.408 |
