# Supplementary material for: Long‐term demography and spatial genetic structure reveal mechanisms of Sassafras albidum population persistence through clonality
Source: Am J Bot. 2026 Jun 7;113(6):e70215. doi: 10.1002/ajb2.70215 (PMC13280968; doi:10.1002/ajb2.70215)
Supplement: Supplementary file 4 — Appendix S4: Spatiotemporal persistence of Sassafras albidum ramet density by size class and census interval using spatial regression with an 8‐nearest‐neighbor spatial weights matrix. [file AJB2-113-e70215-s004.docx]

**Appendix S4.** Spatiotemporal persistence of *Sassafras albidum* ramet density by size class and census interval using spatial regression with an 8-nearest-neighbor spatial weights matrix.

| Size class | Interval | Estimate | Standard error | *t* | *P* | *r*^2^ |
| --- | --- | --- | --- | --- | --- | --- |
| A | 1999-03 | 0.847 | 0.068 | 12.526 | <0.001*** | 0.620 |
|  | 2003-04 | 0.714 | 0.042 | 16.945 | <0.001*** | 0.749 |
|  | 2004-05 | 0.872 | 0.057 | 15.268 | <0.001*** | 0.708 |
|  | 2005-06 | 0.785 | 0.055 | 14.403 | <0.001*** | 0.684 |
|  | 2006-07 | 0.923 | 0.061 | 15.034 | <0.001*** | 0.702 |
|  | 2007-08 | 0.895 | 0.026 | 34.590 | <0.001*** | 0.926 |
|  | 2008-09 | 0.956 | 0.032 | 29.662 | <0.001*** | 0.902 |
|  | 2009-10 | 0.823 | 0.043 | 19.019 | <0.001*** | 0.790 |
|  | 2010-13 | 0.927 | 0.045 | 20.400 | <0.001*** | 0.813 |
|  | 2013-14 | 0.909 | 0.050 | 18.129 | <0.001*** | 0.774 |
|  | 2014-15 | 0.902 | 0.037 | 24.386 | <0.001*** | 0.861 |
|  | 2015-19 | 0.908 | 0.055 | 16.469 | <0.001*** | 0.739 |
|  | 2019-25 | 0.857 | 0.049 | 17.402 | <0.001*** | 0.759 |
| B | 2006-07 | 0.622 | 0.182 | 3.411 | 0.001** | 0.106 |
|  | 2007-08 | 0.679 | 0.072 | 9.416 | <0.001*** | 0.475 |
|  | 2008-10 | 0.582 | 0.123 | 4.717 | <0.001*** | 0.185 |
|  | 2010-19 | 0.319 | 0.073 | 4.355 | <0.001*** | 0.162 |
|  | 2019-25 | 0.603 | 0.086 | 6.993 | <0.001*** | 0.333 |
| C | 2006-07 | -0.103 | 0.122 | -0.844 | 0.401 | 0.007 |
|  | 2007-08 | 0.362 | 0.131 | 2.760 | 0.007** | 0.072 |
|  | 2008-10 | 0.601 | 0.134 | 4.495 | <0.001*** | 0.171 |
|  | 2010-19 | 0.352 | 0.082 | 4.266 | <0.001*** | 0.157 |
|  | 2019-25 | 0.465 | 0.102 | 4.576 | <0.001*** | 0.176 |
| D | 2006-07 | -0.090 | 0.137 | -0.660 | 0.511 | 0.004 |
|  | 2007-08 | -0.099 | 0.113 | -0.884 | 0.379 | 0.008 |
|  | 2008-10 | -0.090 | 0.103 | -0.874 | 0.384 | 0.008 |
|  | 2010-19 | -0.055 | 0.095 | -0.585 | 0.560 | 0.003 |
|  | 2019-25 | 0.365 | 0.075 | 4.832 | <0.001*** | 0.192 |
| E | 2006-07 | 0.593 | 0.048 | 12.243 | <0.001*** | 0.605 |
|  | 2007-08 | 0.613 | 0.060 | 10.276 | <0.001*** | 0.519 |
|  | 2008-10 | 0.291 | 0.070 | 4.157 | <0.001*** | 0.150 |
|  | 2010-19 | -0.022 | 0.078 | -0.287 | 0.775 | 0.001 |
|  | 2019-25 | 0.497 | 0.084 | 5.950 | <0.001*** | 0.265 |
| F | 2006-07 | 0.694 | 0.056 | 12.412 | <0.001*** | 0.611 |
|  | 2007-08 | 0.769 | 0.046 | 16.740 | <0.001*** | 0.741 |
|  | 2008-10 | 0.660 | 0.076 | 8.656 | <0.001*** | 0.433 |
|  | 2010-19 | 0.686 | 0.069 | 9.916 | <0.001*** | 0.501 |
|  | 2019-25 | 0.839 | 0.033 | 25.436 | <0.001*** | 0.868 |
